# Segmental Bioimpedance Variables in Association With Mild Cognitive Impairment

**DOI:** 10.3389/fnut.2022.873623

**Published:** 2022-06-02

**Authors:** Dieu Ni Thi Doan, Boncho Ku, Kahye Kim, Minho Jun, Kyu Yeong Choi, Kun Ho Lee, Jaeuk U. Kim

**Affiliations:** ^1^Department of Digital Health Research, Korea Institute of Oriental Medicine, Daejeon, South Korea; ^2^Korean Convergence Medicine, University of Science and Technology, Daejeon, South Korea; ^3^Gwangju Alzheimer’s Disease and Related Dementias (GARD) Cohort Research Center, Chosun University, Gwangju, South Korea; ^4^Department of Biomedical Science, Chosun University, Gwangju, South Korea; ^5^Dementia Research Group, Korea Brain Research Institute, Daegu, South Korea

**Keywords:** bioelectrical impedance analysis (BIA), segmental analysis, mild cognitive impairment (MCI), Alzheimer’s disease (AD), body composition

## Abstract

**Objective:**

To examine the changes in body composition, water compartment, and bioimpedance in mild cognitive impairment (MCI) individuals.

**Methods:**

We obtained seven whole-body composition variables and seven pairs of segmental body composition, water compartment, and impedance variables for the upper and lower extremities from the segmental multi-frequency bioelectrical impedance analysis (BIA) of 939 elderly participants, including 673 cognitively normal (CN) people and 266 individuals with MCI. Participants’ characteristics, anthropometric information, and the selected BIA variables were described and statistically compared between the CN participants and those with MCI. The correlations between the selected BIA variables and neuropsychological tests such as the Korean version of the Mini-Mental State Examination and Seoul Neuropsychological Screening Battery – Second Edition were also examined before and after controlling for age and sex. Univariate and multivariate logistic regression analyses with estimated odds ratios (ORs) were conducted to investigate the associations between these BIA variables and MCI prevalence for different sexes.

**Results:**

Participants with MCI were slightly older, more depressive, and had significantly poorer cognitive abilities when compared with the CN individuals. The partial correlations between the selected BIA variables and neuropsychological tests upon controlling for age and sex were not greatly significant. However, after accounting for age, sex, and the significant comorbidities, segmental lean mass, water volume, resistance, and reactance in the lower extremities were positively associated with MCI, with ORs [95% confidence interval (CI)] of 1.33 (1.02–1.71), 1.33 (1.03–1.72), 0.76 (0.62–0.92), and 0.79 (0.67–0.93), respectively; with presumably a shift of water from the intracellular area to extracellular space. After stratifying by sex, resistance and reactance in lower extremities remained significant only in the women group.

**Conclusion:**

An increase in segmental water along with segmental lean mass and a decrease in body cell strength due to an abnormal cellular water distribution demonstrated by reductions in resistance and reactance are associated with MCI prevalence, which are more pronounced in the lower extremities and in women. These characteristic changes in BIA variables may be considered as an early sign of cognitive impairment in the elderly population.

## Introduction

Changes in body composition commonly occur with aging in healthy individuals and in those with protein–energy malnutrition, poor physical conditions, or cognitive impairments such as Alzheimer’s disease (AD). Body composition changes over the aging process are characterized by increasing body fat mass, particularly by the reformation of the body’s fat distribution from appendicular fat to trunk fat, with decreasing lean mass and bone mineral density, especially in older adults (1–3). In middle-aged individuals, these changes increase both fat and lean mass, resulting in an increase in body weight (4, 5). Besides these changes, physiological changes in water metabolism and sodium balance occur, leading to alterations in plasma osmolality and changes in the volume within the body’s fluid compartments (6–10).

Mild cognitive impairment (MCI) is an age-related disorder which describes the symptomatic transitional phase between normal cognition and dementia. Clinically, MCI is defined as a set of criteria, including evidence of a change in cognition and lower performance in one or more cognitive domains despite the preservation of independent daily functioning [diagnostic guidelines from National Institute on Aging and the Alzheimer’s Association (11)]. Aside from age, several risk factors contribute to the occurrence and progression of MCI, such as negative lifestyle behaviors and poor dietary habits (12–14). These factors could also lead to chronic non-communicable diseases such as obesity, hypertension, cardiovascular disease, diabetes, and depression, which disrupt physiological homeostasis and hasten the progression of MCI (15, 16).

Recognizing and monitoring changes in body composition can reveal early signs of the body’s vital status, thereby allowing the application of appropriate therapies or lifestyle changes to help reverse the risk of MCI and progression to dementia. Anthropometrical measurements such as weight, height, body mass index (BMI), body circumference, and waist to hip ratio (WHR) have been commonly used in clinical practice to indirectly estimate fat or muscle mass (17–19). However, these measurements can only provide a rough estimate of body mass distribution and cannot discreetly quantify fat or lean mass percentages or intracellular/extracellular water levels (18). Bioelectrical impedance analysis (BIA) is a simple, portable, and relatively accurate technique (20, 21) that can be used to repeatedly measure the body composition of many participants to recognize early changes in body composition status and prognosticate the likelihood of having MCI.

Most previous studies employed specific bioelectrical impedance vector analysis in the later stage of AD rather than during early cognitive impairment. They reported a lower body cell mass, indicated by a lower reactance and phase angle or a shifting to the lower right half of the ellipse, in the AD or dementia group than in individuals with normal cognition (22, 23). These patterns were more prevalent in women with worse psycho-functional status (24, 25). Higher impedance, higher resistance, and longer impedance vector were found in AD patients than in normal controls, suggesting a lower proportion of fat free mass (FFM) (23, 24, 26). Nevertheless, few studies have applied conventional BIA techniques to the assessment of body composition changes in patients with MCI. Some examples include findings of a lower phase angle in women (27) or a higher visceral fat area associated with a lower risk of non-amnestic MCI in older women {mean [standard deviation (SD)] age of 75.0 ± 5.18} (28), though the number of these studies is limited.

Sex is another aspect to be considered for the evaluation and comparison of body composition in individuals with cognitive decline. Sex has a strong relationship with body composition, where women present with a relatively higher fat mass and lower FFM than men (29, 30). Buffa et al. reported a lower body cell mass with dehydration in women with severe AD than in those with mild-moderate AD, but these findings were not evident in men. Regarding bioimpedance, Cova et al. described significantly higher height-normalized values of impedance and resistance in men with MCI than in those without MCI, though this was not observed among women with MCI (26).

Regarding segmental body analysis, few studies have reported changes in the composition of different body segments in individuals with cognitive decline. Mereu et al. demonstrated that bioimpedance results obtained for only the right arm would be similar to whole-body composition in patients with AD (31). Reduction of skeletal muscle mass or muscle strength in the lower limbs was associated with a higher MCI risk in women (32, 33), whereas loss of skeletal muscle mass in the upper and lower limbs was associated with a higher risk of MCI in men (32). Lower extremity skeletal muscle mass was associated with lower cognitive performance and higher cortical beta-amyloid burden (34, 35), whereas upper extremity skeletal muscle mass did not show any association (34). Similarly, lower extremity motor function was found to be prominent in patients with MCI and AD (36, 37).

Subsequently, there is a lack of information about the relationship between body composition, and bioimpedance for each body segment and cognitive decline. Differences by sex in these components between the upper and lower extremities are not well established, especially for individuals with MCI. Therefore, using a segmental multi-frequency BIA technique, we aimed to examine how whole-body composition changes, segmental body fluids, and bioimpedance variables distinctly for the upper and lower extremities differed between cognitively normal (CN) people and those with MCI among both women and men. We hypothesized that lower extremity variables are more strongly linked to the incidence of MCI than upper extremity variables and that this pattern is more noticeable in women than in men. To test our hypotheses, linear logistic regression methods were conducted to examine odds ratios (ORs) before and after controlling for potential covariates, which was then repeated for the different sexes.

## Materials and Methods

### Participants

In total, 1,153 individuals participated in the study. MCI patients were recruited at Chonnam University Hospital (Gwangju City, South Korea) alongside their family members or caregivers, who were recruited as participants with normal cognition. Written informed consent was obtained from each participant or their legal guardian prior to the study. Individuals were excluded from the study if they met one of the following criteria: obtained less than 3 years of education; had a medical history or ongoing acute or chronic illness that interfered with the intended study design, such as neurological diseases, infections, or mental health instability; excessive alcohol consumption; as a CN individual, had a baseline magnetic resonance imaging showing an abnormal pattern or atrophy; and showing clinical signs of hydration imbalance.

**FIGURE 1:**
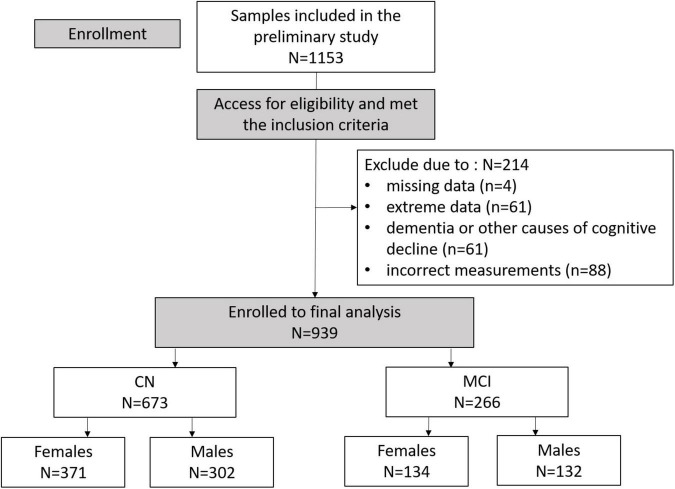
Consolidated Standards of Reporting Trials (CONSORT) diagram illustrating enrollment and exclusion criteria for this study.

Mild cognitive impairment patients were defined as those who were not yet demented, had a Clinical Dementia Rating scale of 0.5, had a Seoul Neuropsychological Screening Battery – Second Edition (SNSB-II) *z*-score no less than −1.5 in at least one of the domains. In contrast, to be considered as CN, participants must have had no history or current symptoms of cognitive decline, a Clinical Dementia Rating of zero, and a SNSB-II *z*-score no less than −1.5 for all tests. All participants aged between 55 and 90 years had adequate hearing and vision on neuropsychological examination. The included participants then underwent a more precise clinical assessment to obtain demographic information and a medical history alongside a general medical examination, neuropsychological tests, and neurophysiological tests. In the data preprocessing, participants who had non-random missing data (*n* = 4), extreme results for any parameters (values above or below three times the interquartile are considered as extreme points) (*n* = 61), and were diagnosed with dementia or other causes of cognitive decline (*n* = 61) were excluded from the analysis. According to the principle of multi-frequency BIA, correct impedance measurements should have lower values for higher frequencies (i.e., impedance at 1 kHz is greater than impedance at 5 kHz); therefore, observations that did not follow this pattern were excluded (*n* = 88). There was a total of 939 participants included in the final analysis, including 673 CN and 266 MCI individuals, as illustrated in Figure 1. The study protocol was approved by the Institutional Review Board of the Chonnam National University Hospital (CNUH; approval number: CNUH-2019-279). The study was performed in agreement with the Declaration of Helsinki.

### Neuropsychological Assessment

The Korean version of the Mini-Mental State Examination (K-MMSE) and SNSB-II were used as neuropsychological assessments in this study.

Seoul Neuropsychological Screening Battery – Second Edition is a comprehensive neuropsychological test that is widely used in Korea for evaluating cognitive performance in people with brain injuries and neurological disorders such as dementia (38, 39). It is composed of five cognitive domains including attention, memory, language, visuospatial function, and frontal/executive function. The attention domain includes the Digit Span Test, vigilance test, and letter cancellation. The memory domain contains the Seoul Verbal Learning Test and the Rey Complex Figure Test. The domain of language and related functions consists of the Boston Naming Test and comprehension, repetition, reading, and writing tests. The visuospatial domain is tested by the Rey Complex Figure Test-Copy and the Clock Drawing Test. The frontal and executive function domain encompasses motor regulation, the Stroop Test, the Controlled Oral Word Association Test, Digit Symbol Coding, and the Trail Making Test. The SNSB-II test and domain scores were normalized by age, sex, and educational levels according to a population 1,067 elderly Korean participants aged 45–90 years who represented normal development (40). The validity and reliability of the SNSB-II has been demonstrated by many previous studies, especially for MCI over other cognitive tests (38).

The K-MMSE is a commonly used screening test for dementia. It tests the five cognitive domains of time and spatial orientation, memory, attention and calculation, language, and visuospatial configuration with a maximum of 30 points.

### Anthropometry and Bioimpedance Measurement

Anthropometric parameters such as height (cm) and weight (kg) were automatically measured from the multi-frequency BIA segmental analyzer Inbody S10 Korea (41) to the nearest 1 mm and 100 g, respectively. BMI was then also auto-calculated (kg/m^2^). Body composition parameters including WHR, FFM, percent body fat (PBF), percent body cell mass (PBCM), total body water per fat free mass (TBW_FFM), and basal metabolic rate (BMR) were estimated from impedance variables obtained from the same device. This device uses a tetrapolar 8-point tactile electrode to measure impedance at six electrical frequencies: 1, 5, 50, 250, 500, and 1,000 kHz and reactance and phase angle at three frequencies: 5, 50, and 250 kHz. Notably, the tetrapolar BIA method was validated as it gives similar results to reference methods such as dual energy X-ray absorptiometry or magnetic resonance imaging techniques (42, 43). The impedance was measured separately for each body segment, including the two arms, two legs, and trunk, producing an estimated segmental body composition and water compartment. Participants were instructed to fast overnight before the examination. Although there are various impedance parameters at several frequencies, this study focused on the impedance variables obtained at 50 kHz. All measurements were carried out by well-trained staff with the Inbody S10 (41) instructions for reference.

### Statistical Analysis

The characteristics of the two groups of participants (CN and MCI) were compared. Demographic information, neuropsychological test results, and body composition characteristics were summarized as means and SDs for continuous variables and as frequencies and proportions for categorical variables. Regarding hypothesizing differences by continuous variables, a univariate independent two-sample *t*-test or Mann–Whitney–Wilcoxon rank sum test was used after confirming distribution normality by the Shapiro–Wilk test. Cohen’s *d* was applied to test the effect size of the difference. To check the independence of each categorical variable with cognitive status, Pearson’s Chi-squared test or Fisher’s exact test was used. Pearson correlation coefficients were employed to examine the bivariate correlations between age, BMI, and neuropsychological test (SNSB-II and K-MMSE) scores with body composition, segmental water, and impedance-related variables. Pearson correlation coefficients were also used to inspect the partial correlations between those variables with conditioning on age and sex, the two factors that are known to be associated with cognition and body composition (44, 45). Univariate and multiple regression analyses were then carried out with cognitive group as the dependent variable and the selected BIA variables as the independent variables, before and after adjusting for the potential confounders. Lastly, we examined the association between MCI and each selected BIA variable after stratifying groups according to sex. A *p*-value of less than 0.05 was considered statistically significant. R version 4.1.2 (The R Project for Statistical Computing^1^) was used for all statistical analyses.

### Covariates

Considering the role of age in the relationship between MCI and changes in body composition and water compartment, we used age as one of the potential confounders in the multivariate regression analysis models in the Tables 3 and 4. As body composition differs between women and men, sex was included as another covariate. Moreover, any significant difference in participants’ basic characteristics that could affect the associations between MCI and body composition variables were incorporated as covariates, including Geriatric Depression Scale (GDS) score, hyperlipidemia, diabetes, and central nervous system disorder.

### Data Selection

Considering that all participants appeared to have a symmetrical body structure upon the in-person screening phase, their left-side was strongly positively correlated with the corresponding variable on the right side of the body for most of the segmental BIA variables (Supplementary Figure 1). The average values between the right and left arm and leg were computed for the upper and lower extremity variables (Supplementary Table 1). The difference between the right and its corresponding left variables produced statistically insignificant results in CN and MCI groups (Supplementary Table 2). In this study, seven whole-body composition variables were employed in the analysis, including BMI, FFM, PBF, PBCM, WHR, TBW_FFM, and BMR. Additionally, seven pairs of segmental body composition, water compartment and bioimpedance variables for the upper and lower extremities were included, consist of segmental water (SW_upper, SW_lower), segmental lean mass (SL_upper, SL_lower), relative water volume to lean mass (Water_Lean_upper, Water_Lean_lower), extracellular to intracellular water ratio (ECW_ICW_upper, ECW_ICW_lower), resistance (R_upper, R_lower), reactance (Xc_upper, Xc_lower), and phase angle (PA_upper, PA_lower). A frequency of 50 kHz was used for all impedance results.

## Results

### Participants’ Characteristics

The demographic information, neuropsychological test scores, and comorbidities for the CN and MCI groups are described in Table 1. Between the CN and MCI groups, there were indifferences in sex, educational years, and blood pressure. Individuals with MCI had a slightly higher mean age than CN participants with the mean (SD) of 73.1 (6.5) and 71.7 (6.2), respectively. The MCI group exhibited poorer cognitive performance with significantly lower SNSB-II and K-MMSE scores and higher levels of depression with an increased GDS score than the CN group. The prevalence of comorbidities including hypertension, thyroid disorders, mental disorders, and circulatory disorders between the groups were equivalent. However, the prevalence of diabetes and CNS disorder were higher and the prevalence of hyperlipidemia was lower in the MCI group when compared with the CN group. For cohabitation, more than 85% of our participants lived with family, and there were no significant differences between the groups.

**TABLE 1:** Demographic information and neuropsychological test results of MCI and CN subjects.

Variables	Total (*n* = 939)^1^	NC (*n* = 673)^1^	MCI (*n* = 266)^1^	*p*-value^2^
Age (year)				**0.002**
Mean (SD)	72.1 (6.3)	71.7 (6.2)	73.1 (6.5)	
Sex, *n* (%)				0.188
Female	505 (53.8%)	371 (55.1%)	134 (50.4%)	
Male	434 (46.2%)	302 (44.9%)	132 (49.6%)	
Education level (year)				0.295
Mean (SD)	12.1 (4.6)	12.0 (4.6)	12.3 (4.4)	
Systolic BP (mmHg)				0.634
Mean (SD)	124 (15)	125 (14)	124 (16)	
Diastolic BP (mmHg)				0.736
Mean (SD)	71 (10)	71 (10)	71 (10)	
GDS score				**0.042**
Mean (SD)	8.3 (6.9)	8.0 (6.8)	9.0 (7.0)	
K-MMSE score				**<0.001**
Mean (SD)	27.2 (2.2)	27.6 (1.9)	26.2 (2.5)	
Attention				**<0.001**
Mean (SD)	9.3 (2.2)	9.7 (2.2)	8.5 (1.8)	
Language				**<0.001**
Mean (SD)	0.2 (0.3)	0.2 (0.3)	0.0 (0.4)	
Visuospatial				**<0.001**
Mean (SD)	0.5 (0.4)	0.5 (0.4)	0.3 (0.6)	
Memory				**<0.001**
Mean (SD)	0.1 (0.7)	0.3 (0.6)	−0.4 (0.6)	
Frontal				**<0.001**
Mean (SD)	0.1 (0.6)	0.2 (0.6)	−0.2 (0.7)	
Diabetes, *n* (%)				**0.011**
Yes	168 (17.9%)	107 (15.9%)	61 (22.9%)	
No	771 (82.1%)	566 (84.1%)	205 (77.1%)	
Hypertension, *n* (%)				0.574
Yes	403 (42.9%)	285 (42.3%)	118 (44.4%)	
No	536 (57.1%)	388 (57.7%)	148 (55.6%)	
Hyperlipidemia, *n* (%)				**0.019**
Yes	183 (19.5%)	144 (21.4%)	39 (14.7%)	
No	756 (80.5%)	529 (78.6%)	227 (85.3%)	
Thyroid, *n* (%)				0.692
Yes	32 (3.4%)	22 (3.3%)	10 (3.8%)	
No	904 (96.6%)	650 (96.7%)	254 (96.2%)	
Mental disorder, *n* (%)				0.627
Yes	21 (2.2%)	14 (2.1%)	7 (2.6%)	
No	917 (97.8%)	658 (97.9%)	259 (97.4%)	
Circulatory disorder, *n* (%)				0.525
Yes	151 (16.1%)	105 (15.6%)	46 (17.3%)	
No	788 (83.9%)	568 (84.4%)	220 (82.7%)	
CNS disorder, *n* (%)				**0.024**
Yes	5 (0.5%)	1 (0.1%)	4 (1.5%)	
No	932 (99.5%)	671 (99.9%)	261 (98.5%)	
Cohabitation, *n* (%)				0.624
Single	126 (13.4%)	88 (13.1%)	38 (14.3%)	
With family	813 (86.6%)	585 (86.9%)	228 (85.7%)	
Dominant hand, *n* (%)				**0.049**
Righty	911 (97.0%)	655 (97.3%)	256 (96.2%)	
Lefty	7 (0.7%)	2 (0.3%)	5 (1.9%)	
Both	21 (2.2%)	16 (2.4%)	5 (1.9%)	

*^1^The values represent mean (SD) for continuous variables, and n (%) for categorical variables. The p-values for the continuous variables were obtained from an independent two sample t-test for normally distributed variables or Mann–Whitney–Wilcoxon rank sum test for non-normally distributed variables. For the categorical variables, the p-values were derived from the Chi-squared test statistics or Fisher-exact test. CNS, central nervous system.*

*^2^Wilcoxon rank sum test; Pearson’s Chi-squared test; two sample t-test; Fisher’s exact test. The bold fonts indicate a p-value lower than 0.05.*

### Anthropometric and Whole-Body Composition Results

Anthropometric information and the whole-body composition results were described in Table 2. In terms of anthropometric indices, the CN and MCI groups had comparable height, weight, and therefore BMI. However, MCI participants had a slightly lower body fat indices as indicated by lower PBF with a mean (SD) of 32.1 (7.6) and 33.3 (7.9) and smaller WHR with a mean (SD) of 0.879 (0.063) and 0.887 (0.055) compared with CN individuals, respectively. Similarly, MCI participants had a slightly higher PBCM with respect to CN individuals with a mean (SD) of 43.5 (5.0) and 42.9 (5.2) and greater TWB_FFM with those of 73.92 (0.29) and 73.88 (0.27), respectively.

**TABLE 2:** Anthropometry and whole-body composition results.

Variables	Total (*n* = 939)^1^	CN (*n* = 673)^1^	MCI (*n* = 266)^1^	*p*-value^2^
Height (cm)				0.162
Mean (SD)	159.4 (8.4)	159.2 (8.3)	159.9 (8.5)	
Weight (kg)				0.977
Mean (SD)	62.7 (9.5)	62.7 (9.3)	62.7 (10.1)	
BMI (kg/m^2^)				0.111
Mean (SD)	24.6 (2.9)	24.7 (2.8)	24.4 (3.0)	
FFM (kg)				0.228
Mean (SD)	42.0 (7.9)	41.8 (7.9)	42.4 (8.0)	
PBF (%)				**0.018**
Mean (SD)	33.0 (7.8)	33.3 (7.9)	32.1 (7.6)	
PBCM (%)				**0.044**
Mean (SD)	43.1 (5.2)	42.9 (5.2)	43.5 (5.0)	
WHR				**0.028**
Mean (SD)	0.885 (0.057)	0.887 (0.055)	0.879 (0.063)	
TBW_FFM				**0.038**
Mean (SD)	73.89 (0.28)	73.88 (0.27)	73.92 (0.29)	
BMR (kcal)				0.226
Mean (SD)	1,277 (171)	1,273 (171)	1,287 (172)	

*^1^The values represent mean (SD) for continuous variables, and n (%) for categorical variables. The p-values for the continuous variables were obtained from an independent two sample t-test for normally distributed variables or Mann–Whitney–Wilcoxon rank sum test for non-normally distributed variables. For the categorical variables, the p-values were derived from the Chi-squared test statistics or Fisher-exact test.*

*^2^Wilcoxon rank sum test. The bold fonts indicate a p-value lower than 0.05.*

### Descriptions of the Segmental Body Composition and Bioimpedance Variables

**FIGURE 2:**
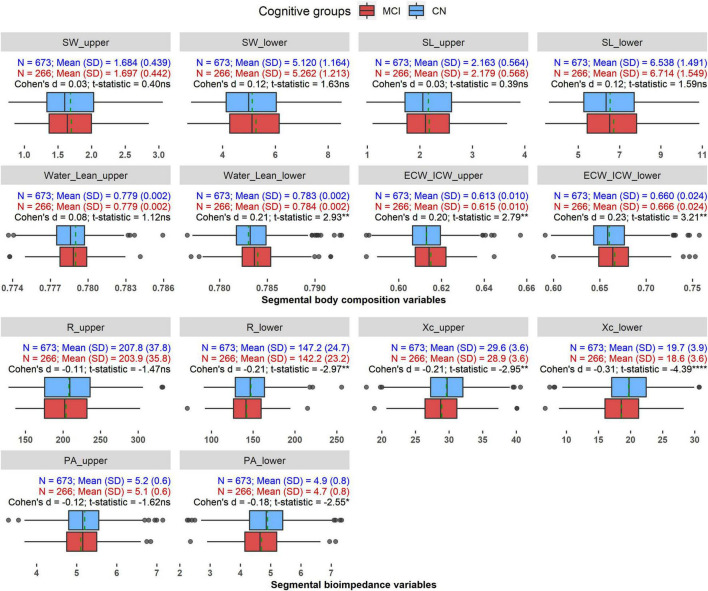
The distributions of the segmental BIA variables in CN and MCI groups. *p < 0.05, **p < 0.01, ***p < 0.0001, ns: Not significant. Dashed green line indicates mean value of each variable in each group.

Figure 2 visualizes the data of the selected segmental variables and comparative statistics between the two groups. Among the segmental body composition and water compartment variables, the MCI group exhibited a tendency of increasing relative water volume and extra- to intracellular water ratios compared to CN group indicated by significant effect size of approximately 0.20 for Water_Lean_lower, ECW_ICW_upper, and ECW_ICW_lower. For the segmental bioimpedance variables, resistance, reactance, and phase angle displayed a similar pattern of decreasing in the MCI group when compared with the CN group, although resistance and phase angle showed significant differences between the two groups only for the lower extremity (R_lower and PA_lower). The effect sizes in these impedance comparisons ranged from −0.18 to −0.31.

### Correlations Between Age, Neuropsychological Test Scores, Body Composition, Water Compartment, and Bioimpedance Variables

**FIGURE 3:**
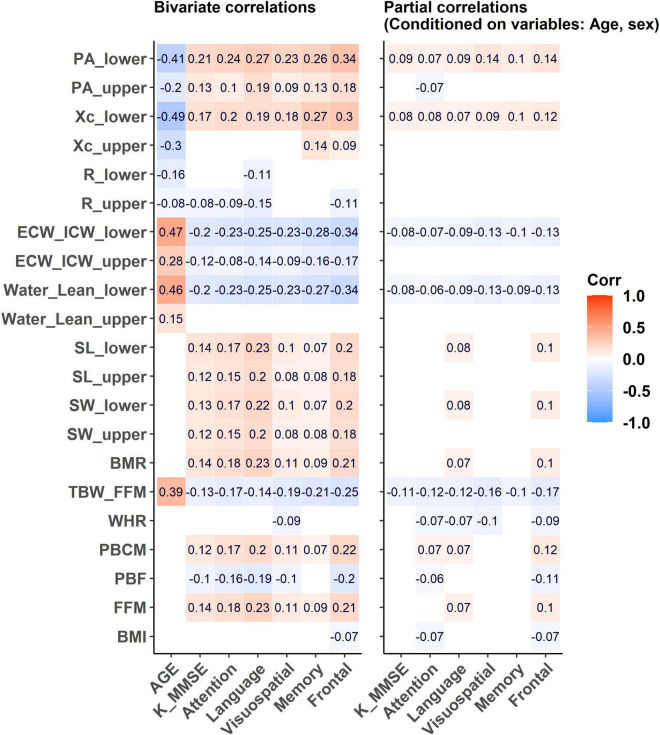
The correlations between age and cognitive tests such as SNSB-II domains and K-MMSE score with the selected BIA variables and its partial correlations after controlling for age and sex. Empty cells: insignificant correlation coefficients (*p*-value > 0.05).

Figure 3 illustrates the correlations between age and the cognitive tests with the selected BIA variables, as well as partial correlations after controlling for age and sex.

#### Bivariate Correlations

##### Age

Age displayed positive correlations with the water-related variables but negative correlations with the impedance-related variables. Age demonstrated relatively stronger correlations with the lower extremity variables such as Water_Lean_lower, ECW_ICW_lower, Xc_lower, and PA_lower than the corresponding upper extremity variables. With aging, relative water volume (Water_Lean_lower, *r* = 0.46) and cellular water ratio (ECW_ICW_lower, *r* = 0.47) variables in the lower extremity tended to increase. The reactance and phase angle variables in the lower extremity tended to decrease with aging (Xc_lower, *r* = −0.49; PA_lower, *r* = −0.41). Whole-body water hydration status indicated by the ratio of TBW_FFM was positively correlated with age (*r* = 0.39), whereas other parameters such as PBF, PBCM, and BMR did not show significant correlations.

##### Neuropsychological Tests

All BIA variables had weak or no correlations with the five domains of the SNSB-II and the K-MMSE. The highest coefficients were found between the SNSB-II frontal domain and Water_Lean_lower, ICW_ECW_lower, Xc_lower, and PA_lower in the lower extremity, with a maximum coefficient of 0.34 (absolute value). K-MMSE had the highest positive correlation with PA_lower, with a coefficient of 0.21. Overall, the correlations were more observable with lower extremity variables than with those of the upper extremity.

#### Partial Correlations

After adjusting for age and sex, most of the significant correlations between the BIA variables and the neuropsychological test scores were diminished. The highest coefficients were obtained between the SNSB-II visuospatial and frontal domains and PA_lower with *r* = 0.14 and TBW_FFM with *r* = −0.16 and *r* = −0.17, respectively. These results determined the role of these covariates in the association between BIA variables and cognitive tests.

### Relationship Between the Bioelectrical Impedance Analysis Variables and Mild Cognitive Impairment

Table 3 presents the relationship between each of the BIA variables and MCI prevalence by ORs obtained from univariate and multiple logistic regression before and after adjusting for the potential covariates.

**TABLE 3:** Estimated odds ratios and 95% confidence intervals derived from the two logistic regression models.

	Crude model			Adjusted model		
Variables	OR^1^	95% CI^1^	*p*-value^2^	OR^1^	95% CI^1^	*p*-value^2^
BMI	0.91	0.79, 1.05	0.196	0.94	0.81, 1.09	0.430
FFM	1.08	0.94, 1.24	0.282	1.20	0.92, 1.57	0.173
PBF	0.86	0.75, 0.99	**0.041**	0.84	0.70, 1.02	0.072
PBCM	1.13	0.98, 1.31	0.088	1.17	0.96, 1.42	0.111
WHR	0.86	0.74, 0.99	**0.033**	0.87	0.75, 1.01	0.067
TBW_FFM	1.17	1.02, 1.35	**0.028**	1.09	0.92, 1.27	0.319
BMR	1.08	0.94, 1.24	0.282	1.20	0.92, 1.57	0.174
SL_upper	1.03	0.89, 1.18	0.699	1.02	0.79, 1.32	0.856
SL_lower	1.12	0.97, 1.29	0.108	1.32	1.02, 1.71	**0.033**
SW_upper	1.03	0.89, 1.19	0.690	1.03	0.79, 1.32	0.847
SW_lower	1.13	0.98, 1.30	0.098	1.33	1.03, 1.72	**0.031**
Water_Lean_upper	1.09	0.94, 1.25	0.254	1.04	0.90, 1.20	0.599
Water_Lean_lower	1.23	1.07, 1.42	**0.004**	1.14	0.96, 1.35	0.140
ECW_ICW_upper	1.22	1.06, 1.41	**0.006**	1.15	0.99, 1.35	0.068
ECW_ICW_lower	1.26	1.09, 1.45	**0.001**	1.17	0.99, 1.39	0.067
R_upper	0.90	0.78, 1.04	0.149	0.84	0.65, 1.08	0.163
R_lower	0.81	0.70, 0.93	**0.004**	0.76	0.62, 0.92	**0.005**
Xc_upper	0.81	0.70, 0.93	**0.003**	0.86	0.73, 1.01	0.064
Xc_lower	0.73	0.63, 0.85	**<0.001**	0.79	0.67, 0.93	**0.005**
PA_upper	0.89	0.77, 1.03	0.109	0.88	0.73, 1.06	0.193
PA_lower	0.83	0.72, 0.96	**0.012**	0.89	0.74, 1.06	0.182

*^1^OR, odds ratio; CI, confidence interval.*

*^2^p-value obtains from Wald test. The models adjusted for age, sex, GDS score, hyperlipidemia, diabetes, and CNS disorder. The bold fonts indicate a p-value lower than 0.05.*

Before controlling for the covariates, several BIA variables showed significant associations with MCI, such as the whole-body composition variables PBF, WHR, and TBW_FFM or the segmental variables Water_Lean_lower, ECW_ICW_upper, ECW_ICW_lower, R_lower, Xc_upper, Xc_lower, and PA_lower, with significant ORs and their 95% confidence intervals (CIs) away from 1. Regarding whole-body composition, PBF and WHR exhibited negative associations with MCI, whereas TBW_FFM had a positive association with MCI with ORs (95% CI) of 0.86 (0.75–0.99), 0.86 (0.74–0.99), and 1.17 (1.02–1.35), respectively. With respect to segmental variables, there was a positive association between the water-related variables and MCI, and a negative association between the impedance-related variables and MCI. A unit increase in Water_Lean_lower, ECW_ICW_upper, or ECW_ICW_lower was associated with a nearly 22–26% increase in the odds of having MCI, whereas one unit decrease in R_lower, Xc_upper, Xc_lower, or PA_lower was associated with an approximate 17–27% increase in the odds of having MCI.

After adjusting for age, sex, GDS score, and the comorbidities, PBF, WHR, and TBW_FFM were no longer associated with MCI according to their *p*-values. R_lower and Xc_lower remained associated with MCI prevalence, with ORs and 95% CIs of 0.76 (0.62–0.92) and 0.79 (0.67–0.93), respectively; decreasing one unit in one of these variables was associated with increasing the odds of MCI by 21–24%. Noticeably, SW_lower and SL_lower which did not associate with MCI in the crude model became significantly associated with MCI after adjusting for the covariates, with the OR (95% CIs) of 1.32 (1.02–1.71) and 1.33 (1.03–1.72), respectively. Cellular water distribution between extra- and intra-spaces indicated by ECW_ICW_upper and ECW_ICW_lower variables exhibited a tendency of negatively associated with MCI which was demonstrated by an adjoining statistical significance threshold of the ORs (approximate *p*-value = 0.07). Relative water volume in both upper and lower extremities (Water_Lean_upper, Water_Lean_lower) did not show significant associations with the incidence of MCI.

### Relationship Between the Bioelectrical Impedance Analysis Variables and Mild Cognitive Impairment for Each Sex

Table 4 separately examined the relationship between the BIA variables and MCI for women and men. After stratifying by sex, R_lower and Xc_lower were significantly associated with MCI in women, with ORs (95% CI) of 0.74 (0.57–0.95) and 0.75 (0.60–0.94), respectively. There were no significant associations between MCI and any of the BIA variables in the men group.

**TABLE 4:** Estimated odds ratios and 95% confidence intervals derived from the logistic regression models in women and men.

	Women			Men		
Variables	OR^1^	95% CI^1^	*p*-value^2^	OR^1^	95% CI^1^	*p*-value^2^
BMI	0.92	0.75, 1.11	0.387	1.01	0.80, 1.28	0.906
FFM	1.25	0.80, 1.96	0.320	1.24	0.88, 1.74	0.217
PBF	0.78	0.61, 1.01	0.062	0.92	0.70, 1.22	0.572
PBCM	1.26	0.97, 1.65	0.088	1.07	0.80, 1.42	0.654
WHR	0.85	0.69, 1.05	0.139	0.89	0.72, 1.09	0.270
TBW_FFM	1.10	0.88, 1.37	0.389	1.06	0.83, 1.35	0.650
BMR	1.26	0.80, 1.97	0.313	1.23	0.88, 1.73	0.221
SL_upper	1.05	0.68, 1.60	0.831	1.06	0.76, 1.48	0.714
SL_lower	1.32	0.86, 2.04	0.199	1.36	0.98, 1.89	0.066
SW_upper	1.05	0.69, 1.60	0.827	1.07	0.77, 1.48	0.706
SW_lower	1.33	0.87, 2.05	0.189	1.36	0.98, 1.88	0.064
Water_Lean_upper	1.01	0.84, 1.22	0.910	1.08	0.86, 1.36	0.517
Water_Lean_lower	1.21	0.95, 1.55	0.116	1.07	0.84, 1.36	0.594
ECW_ICW_upper	1.19	0.94, 1.51	0.140	1.12	0.91, 1.38	0.277
ECW_ICW_lower	1.23	0.96, 1.57	0.100	1.13	0.89, 1.43	0.326
R_upper	0.79	0.57, 1.08	0.146	0.87	0.56, 1.32	0.508
R_lower	0.74	0.57, 0.95	**0.020**	0.75	0.55, 1.02	0.071
Xc_upper	0.81	0.64, 1.01	0.061	0.91	0.71, 1.15	0.412
Xc_lower	0.75	0.60, 0.94	0.014	0.81	0.63, 1.04	0.101
PA_upper	0.88	0.67, 1.16	0.380	0.91	0.70, 1.18	0.481
PA_lower	0.86	0.66, 1.12	0.255	0.92	0.71, 1.17	0.487

*^1^OR, odds ratio; CI, confidence interval.*

*^2^p-value obtains from Wald test. The models adjusted for age, GDS score, hyperlipidemia, diabetes, and CNS disorder. The bold fonts indicate a p-value lower than 0.05.*

## Discussion

In this study, we aimed to examine the association between the BIA variables with the incidence of MCI, including seven whole-body composition variables and seven pairs of segmental BIA variables. The MCI group was shown to be slightly older, more depressive, and had significantly poorer cognitive abilities than CN group. The CN and MCI groups had approximately equivalent body anthropometric indices such as height, weight, and BMI. However, individuals with MCI had a slightly lower fat mass as indicated by lower WHR and PBF and higher PBCM and TBW_FFM than the CN participants (Table 2).

Segmental analysis revealed that MCI individuals had a higher amount of relative water volumes and extra- to intracellular water ratios, but lower resistance, reactance, and phase angle in the upper and/or lower extremities when compared with CN individuals (Figure 2). After accounting for age, sex, GDS score, and the comorbidities, although the whole-body composition variables failed to differentiate CN and MCI, segmental analysis found that segmental water, segmental lean mass, resistance and reactance variables of the lower extremity were significantly associated with MCI (Table 3).

First of all, the segmental water and segmental lean mass in the lower extremities were proportionally associated with MCI, but their ratio indicated by the Water_Lean_lower variable was not significantly associated with MCI (Table 3). This result suggests that the increase in segmental water was directly proportional to the increase in segmental lean mass in the lower extremity, reflecting an increase in segmental lean mass but equal water volume per lean mass in the cognitive decline subjects. Despite the water volume normality, the water distribution between extra- and intracellular spaces showed the tendency of a shifting of water from the intracellular space to the extracellular areas, demonstrating by the ORs of ECW_ICW_lower closely adjoin to the statistical significance threshold (Table 3). This tendency suggests an abnormal water distribution and cellular dehydration status in individuals with cognitive decline, independently of age and the other covariates.

Secondly, the reduction in lower extremity reactance was found to be associated with the higher prevalence of MCI, independent of age, sex, and the other covariates. Lower reactance indicates a lower cell membrane capacitance due to a reduction in body cell mass or a decrease in cell strength due to malnutrition (20, 46). Cova et al. reported a lower nutritional status as indicated by a lower Mini Nutritional Assessment score and a decrease in body cell mass as demonstrated by a shifting of the RXc graph on the right side in individuals with MCI when compared with the reference (26). In studies of dementia, lower reactance was found in women with severe AD than in patients with mild-to-moderate AD, as it was more evident in women with worse psycho-functional status (24, 25) and in men with dementia (23). Our finding of a reactance reduction in the MCI group is in alignment with these publications as we found a negative association between reactance and MCI, demonstrating a decrease in body cell mass or cell strength in the lower extremity in the individuals with cognitive decline. However, considering the positive association between SL_lower and MCI which implies an increase of segmental lean mass in lower extremity, the decrease in body cell mass might be due to the reduction in the volume of individual cell rather than decrease in total cell quantity. Most of living cells contain a large amount of water (47), so that ICW accounts for approximately 70% of total cell mass (48). The decrease in body cell mass or presumably reduced volume of individual cell therefore might be due to the reduction of ICW content in the MCI individuals.

Furthermore, resistance in lower extremity was also found to be linked with the higher prevalence of MCI, regardless of age and the other covariates. Resistance is a measure of resistivity and is inversely proportional to the relative amount of total body water, decrease in resistance indicates a higher proportion of water volume (20, 49). Saragat et al. reported a higher height-normalized resistance in both sexes with AD, but a preserved value in patients with lower psychological status (MMSE < 20.7 compared with MMSE ≥ 20.7 in CN individuals), suggesting a relative increase in fat in individuals with AD than that in CN individuals (24). The same team reported dehydration in women with severe AD as showed by a lengthening of the impedance vector on the RXc graph compared to the control (25). In this study, our MCI group did not have an increase in fat mass percentage or dehydration. In contrast, they had a higher segmental lean mass and a tendency of higher extracellular water compared with CN individuals (Figure 2 and Table 3). Consequently, the increase in segmental lean mass and ECW volume might cause the R_lower reduction in the MCI individuals.

In this study, those with MCI were in the early stage of cognitive decline, with a mean (SD) K-MMSE score of 26.2 (2.5) that is somewhat closer to the mean (SD) of 27.6 (1.9) seen in the CN group than those in the aforementioned studies. Similarly, our MCI participants had better cognitive test scores than the cognitively impaired individuals in the previous studies (24, 25). Therefore, they might not yet have substantial changes in physical activity or nourishment and thus did not experience solid changes in body composition. Furthermore, age was reported to be an important factor to influence the fluctuation of cellular water distribution, such that ECW to ICW ratio increases with age and might be due to the steeper decrease in the ICW content than in the ECW compartment (9, 50). After controlling for age and other covariates, most body composition variables became insignificant; only segmental BIA variables such as resistance and reactance in the lower extremities and the related body composition/water compartment variables such as SL_lower and SW_lower remained to be significantly associated with the incidence of MCI.

After stratifying by sex, we found that women with MCI showed a significant reduction in terms of reactance and resistance of the lower extremity, whereas this pattern did not emerge in men (Table 4). The body composition differences between men and women were diversely reported in previous BIA publications (22, 24, 28). Sex hormones have been known to strongly influence body fat distribution, particularly estrogen and progesterone in women during menopause (49, 51). A common finding is that there is a higher possibility of obesity with predominant abdominal fat accumulation in aging women with a decrease in estrogen secretion (52, 53). However, Collelueri et al. described a U-shaped relationship between estradiol (a major estrogen in females) and body fat mass, such that total body fat mass was found to be decreased when estradiol levels were between 14.0 and 17.4 pg/ml and increased elsewhere (54). The complex relationship between hormonal changes during aging and body composition might regulate the relationship between impedance variables and cognitive decline. Nonetheless, our findings suggest that women with MCI experience more dramatic changes in their body composition and water compartment than men with MCI.

In this study, whole-body composition measurements, such as FFM or PBCM, were investigated as indicators of muscle volume in relation to MCI. Although individuals with MCI showed slightly more muscle volume due to larger FFM and PBCM compared with CN individuals (Table 2), these variables were insignificantly linked with MCI when taking into account age, sex, GDS score, and comorbidities (Table 3). However, segmental reactance in the lower extremity, which is associated with segmental cell mass or segmental muscle volume was significantly associated with the incidence of MCI, especially in women (Tables 3, 4). Individuals with cognitive impairment are likely to experience protein–energy malnutrition and/or decreased physical activity, thus having a decrease in body lean mass or muscle volume. Myokines, which are synthesized and secreted from muscle mass during muscular contractions, regulate the metabolism in the muscle itself (autocrine) as well as in other tissues and organs, such as the brain, through their receptors (para/endocrine) (55). A lower muscle volume reduces myokine secretion and therefore lowers the amount of myokines crossing the blood–brain barrier. This imbalance in the myokine level upregulates pro-inflammatory cytokine production and impairs glucose and lipid metabolism in the brain (56). As a result, individuals with lower muscle volume or muscle atrophy are susceptible to neuronal damage which induces abnormal cognitive ability (57). In this study, a significant reduction in lower extremity reactance indicating a lower body cell mass or lower muscle volume and therefore a decrease in myokine secretion, might be one of the causes of cognitive impairment in the individuals with MCI (58). This finding revealed the importance of segmental body cell mass or muscle volume when examining the association with cognitive decline, especially in women (59).

In terms of muscle quality, phase angle and ECW/ICW have been recommended as indicators of muscle quality (60, 61). Phase angle is a major parameter previously investigated as a predictor of morbidity and mortality for several pathological conditions or critical illnesses, such as mortality in intensive care or in postoperative patients (62–64), sarcopenia, frailty (65), and Human Immunodeficiency Virus infection (66). It is also suggested as a potential indicator for diabetes (67), hyperlipidemia (68), and hypertension (69), which are known risk factors of cognitive impairment. The phase angle cutoff threshold varies across samples of different ages, sexes, and morbidities (65, 70, 71). In this study, MCI patients of both sexes had just a slightly lower phase angle than the normal controls, and this difference disappeared after controlling for covariates in both women and men; the muscle quality expressed by phase angle might not be a sensitive marker of cognitive impairment in its early stage. Moreover, ECW/ICW is another sensitive parameter that is positively correlated with age as well as cognitive function such as executive function, inhibition, or attention ability in elderly people (9, 50). In this study, after controlling for age, sex and the comorbidities, ECW/ICW in upper and lower extremities showed a tendency of associations with MCI prevalence. Further investigations with individuals in the early cognitive impairment stages are needed to confirm the relation between ECW/ICW and MCI.

In the correlation analysis, age was the primary factor in the correlations between cognitive tests (SNSB-II and K-MMSE) and the BIA variables (Figure 3). After adjusting for age and sex, the cognitive tests showed only weak correlations with some BIA variables; resistance and reactance variables did not or only weakly correlated with the cognitive test scores. However, it was still able to produce significant associations with the incidence of MCI, regardless of age and the other covariates. Since the cognitive impairment in the older individuals is a degenerative symptom, it seems to be accompanied by overall decline in physiological functions with an early change of cellular water volume especially in lower extremity.

Daily activities involve physical strengths in both arms and both legs. In this study, bioimpedance changes in the lower extremity were observed and were more significantly associated with MCI than those in the upper extremity. A reduction in physical function in the lower extremity is likely to be related to a higher risk of cognitive decline (32, 33); it suggests that more focus should be placed on impedance variable changes in the lower extremity in relation to the early stage of cognitive impairment.

In this study, among multiple frequencies of 5, 50, and 250 kHz, the frequency of 50 kHz was selected to simplify the data analysis and be representative of the impedance variables. Indeed, the bioimpedance variables at other frequencies, such as 5 and 250 kHz, behave similarly as those at 50 kHz; lower extremity resistance and reactance variables at those frequencies were associated with MCI prevalence as well (Supplementary Table 4).

Compared to precedent publications (22–27, 36), our study contained a sufficient sample size of MCI and CN participants and thus can provide additional insight into body composition/impedance changes occurring in the early stage of cognitive decline. Regarding the transition from CN to dementia, MCI individuals represented a closer-to-normal stage based on cognition test scores. Despite this limitation, segmental bioimpedance variables such as resistance and reactance in the lower extremity and the related water compartment/body composition components were still effective in recognizing and distinguishing MCI patients from CN individuals. These lower extremity features might be used as an early sign of cognitive decline.

There are some limitations in this study. First, confounding factors, such as daily activity hours and nutritional states, may affect body composition and bioimpedance characteristics (72, 73). Second, segmental impedance variables were derived directly from BIA measurements, whereas body composition and segmental water compartments were estimated based on these impedance variables; therefore, accuracy may vary between ethnicity, age, sex, and manufacturer. Third, longitudinal studies are needed to monitor and confirm the changes in body composition, water compartment and bioimpedance features even during the earlier stages of cognitive decline (e.g., from CN or subjective memory impairment).

## Conclusion

The selected BIA variables, which reflect the changes in body composition, water compartment and bioimpedance for upper and lower extremities, have weak correlations with the K-MMSE score as well as with the SNSB-II domains, especially when taking age and sex into consideration. Nevertheless, multiple logistic regression analysis reveals significant associations between the lower extremity BIA variables with MCI. Specifically, we found an increase in segmental lean mass and a decrease in body cell volume due to an abnormal cellular water distribution in the lower extremities with MCI, which were demonstrated by the reductions in both resistance and reactance. These characteristic changes of BIA variables may be considered as an early sign accompanied by cognitive impairment in the older population.

## Data Availability Statement

The original contributions presented in the study are included in the article/Supplementary Material, further inquiries can be directed to the corresponding author.

## Ethics Statement

The studies involving human participants were reviewed and approved by the Institutional Review Board of the Chonnam National University Hospital. The patients/participants provided their written informed consent to participate in this study.

## Author Contributions

DD analyzed the data and wrote the manuscript. BK assisted in the data analysis. KK handled the Institutional Review Board approval and managed the data. MJ participated in writing the manuscript. KC and KL took care of the data collection and curation. JK designed the study and wrote the manuscript. All authors revised and approved the contents of the manuscript, contributed to the article, and approved the submitted version.

## Conflict of Interest

The authors declare that the research was conducted in the absence of any commercial or financial relationships that could be construed as a potential conflict of interest.

## Publisher’s Note

All claims expressed in this article are solely those of the authors and do not necessarily represent those of their affiliated organizations, or those of the publisher, the editors and the reviewers. Any product that may be evaluated in this article, or claim that may be made by its manufacturer, is not guaranteed or endorsed by the publisher.

## Footnotes

1
http://www.r-project.org/
