# Corrigendum: Segmental bioimpedance variables in association with mild cognitive impairment

**DOI:** 10.3389/fnut.2022.1006423

**Published:** 2022-09-14

**Authors:** Dieu Ni Thi Doan, Boncho Ku, Kahye Kim, Minho Jun, Kyu Yeong Choi, Kun Ho Lee, Jaeuk U. Kim

**Affiliations:** ^1^Department of Digital Health Research, Korea Institute of Oriental Medicine, Daejeon, South Korea; ^2^Korean Convergence Medicine, University of Science and Technology, Daejeon, South Korea; ^3^Gwangju Alzheimer's Disease and Related Dementias (GARD) Cohort Research Center, Chosun University, Gwangju, South Korea; ^4^Department of Biomedical Science, Chosun University, Gwangju, South Korea; ^5^Dementia Research Group, Korea Brain Research Institute, Daegu, South Korea

**Keywords:** bioelectrical impedance analysis (BIA), segmental analysis, mild cognitive impairment (MCI), Alzheimer's disease (AD), body composition

In the published article, there was an error in **Supplementary Table S1**. The definition of R_upper and R_lower were incorrectly described. The corrected table can be found in the **Supplementary Material** in the original article.

The authors apologize for this error and state that this does not change the scientific conclusions of the article in any way. The original article has been updated.

## Publisher's note

All claims expressed in this article are solely those of the authors and do not necessarily represent those of their affiliated organizations, or those of the publisher, the editors and the reviewers. Any product that may be evaluated in this article, or claim that may be made by its manufacturer, is not guaranteed or endorsed by the publisher.

