# Supplementary material for: Segmental Bioimpedance Variables in Association With Mild Cognitive Impairment
Source: Front Nutr. 2022 Jun 2;9:873623. doi: 10.3389/fnut.2022.873623 (PMC9201435; doi:10.3389/fnut.2022.873623)
Supplement: Supplementary file 1 [file Table_1.docx]

Supplementary Materials

**Table S1**. Description of the segmental variables considered in this study.

| **Variable** | **Description** | **Unit** | **Definition** |
| --- | --- | --- | --- |
| SW_upper | Average total segmental water in upper limbs | L | (SW_RA + SW_LA)/2 |
| SW_lower | Average total segmental water in lower limbs | L | (SW_RL + SW_LL)/2 |
| SL_upper | Average segmental lean in upper limbs | Kg | (SL_RA + SL_LA)/2 |
| SL_lower | Average segmental lean in lower limbs | Kg | (SL_RL + SL_LL)/2 |
| Water_Lean_upper | Ratio of average segmental water to average segmental lean in upper limbs | _ | $\frac{SW\_RA+SW\_LA}{SL\_RA+SL\_LA}$ |
| Water_Lean_lower | Ratio of average segmental water to average segmental lean in lower limbs | _ | $\frac{SW\_RL+SW\_LL}{SL\_RL+SL\_LL}$ |
| ECW_ICW_upper | Average of segmental extra-intracellular water ratio in upper limbs | _ | ($\frac{ECW\_TBW\_RA}{1-ECW\_TBW\_RA}$ + $\frac{ECW\_TBW\_LA}{1-ECW\_TBW\_LA}$)/2 |
| ECW_ICW_lower | Average of segmental extra-intracellular water ratio in lower limbs | _ | ($\frac{ECW\_TBW\_RL}{1-ECW\_TBW\_RL}$ + $\frac{ECW\_TBW\_LL}{1-ECW\_TBW\_LL}$)/2 |
| R_upper | Average of segmental resistance in upper limbs at 50 kHz normalized by height | Ohm/m | $\frac{\sqrt{{Z\_RA}^{2}-{Xc\_RA}^{2}}+\sqrt{{Z\_LA}^{2}-{Xc\_LA}^{2}}}{2}$/Height |
| R_lower | Average of segmental resistance in lower limbs at 50 kHz normalized by height | Ohm/m | $\frac{\sqrt{{Z\_RL}^{2}-{Xc\_RL}^{2}}+\sqrt{{Z\_LL}^{2}-{Xc\_LL}^{2}}}{2}$/Height |
| Xc_upper | Average of segmental reactance in upper limbs at 50 kHz | Ohm | $\frac{Xc\_RA+Xc\_LA}{2}$ |
| Xc_lower | Average of segmental reactance in lower limbs at 50 kHz | Ohm | $\frac{Xc\_RL+Xc\_LL}{2}$ |
|  | Phase angle at 50 kHz | Degree | tan^-1^ ($\frac{\mathrm{Xc}}{sqrt(Z^2-Xc^2)})*180/\pi$ |
| PA_upper | Average of segmental phase angle in upper limbs at 50 kHz | Degree | $\frac{PA\_RA+PA\_LA}{2}$ |
| PA_lower | Average of segmental phase angle in lower limbs at 50 kHz | Degree | $\frac{PA\_RL+PA\_LL}{2}$ |
| RA: Right arm, LA: Left arm, RL: Right leg, LL: Left leg, SW: Segmental water, SL: Segmental lean, ICW: Intracellular water, ECW: Extracellular water, TBW: Total body water, Z: Impedance, Xc: Reactance, R: Resistance, PA: Phase angle – All of these parameters were directly derived from InbosyS10. | | | |

**Table S2.** Differences between left and right in the segmental body composition and bioimpedance variables between CN and MCI groups

| **Variables** | **Total (n = 939)^1^** | **CN (n=673)^1^** | **MCI (n=266)^1^** | **p_value^2^** |
| --- | --- | --- | --- | --- |
| **SW_upper_diff** | 0.01 (-0.02, 0.05) | 0.02 (-0.02, 0.05) | 0.01 (-0.01, 0.04) | 0.751 |
| **SW_lower_diff** | 0.03 (-0.03, 0.11) | 0.03 (-0.03, 0.10) | 0.03 (-0.03, 0.12) | 0.770 |
| **SL_upper_diff** | 0.02 (-0.02, 0.06) | 0.02 (-0.02, 0.06) | 0.02 (-0.02, 0.06) | 0.687 |
| **SL_lower_diff** | 0.05 (-0.04, 0.15) | 0.05 (-0.03, 0.14) | 0.04 (-0.04, 0.15) | 0.860 |
| **Water_Lean_upper_diff** | 0.00 (0.00, 0.00) | 0.00 (0.00, 0.00) | 0.00 (0.00, 0.00) | 0.780 |
| **Water_Lean_lower_diff** | 0.00 (0.00, 0.00) | 0.00 (0.00, 0.00) | 0.00 (0.00, 0.00) | 0.300 |
| **ECW_ICW_upper_diff** | 0.00 (-0.01, 0.00) | 0.00 (-0.01, 0.00) | 0.00 (-0.01, 0.00) | 0.988 |
| **ECW_ICW_lower_diff** | -0.01 (-0.01, 0.00) | -0.01 (-0.01, 0.00) | -0.01 (-0.01, 0.00) | 0.731 |
| **R_upper_diff** | -1.85 (-6.01, 2.79) | -1.99 (-6.30, 2.91) | -1.52 (-5.26, 2.46) | 0.467 |
| **R_lower_diff** | -1.18 (-5.32, 2.33) | -1.27 (-5.21, 2.31) | -1.15 (-5.63, 2.36) | 0.892 |
| **Xc_upper_diff** | 0.10 (-0.70, 1.00) | 0.10 (-0.70, 1.00) | 0.00 (-0.62, 0.90) | 0.899 |
| **Xc_lower_diff** | 0.15 (-0.80, 1.00) | 0.20 (-0.80, 1.10) | 0.10 (-0.80, 0.90) | 0.308 |
| **PA_upper_diff** | 0.10 (-0.10, 0.20) | 0.10 (-0.10, 0.20) | 0.10 (-0.10, 0.20) | 0.565 |
| **PA_lower_diff** | 0.10 (-0.10, 0.30) | 0.10 (-0.10, 0.30) | 0.10 (-0.10, 0.30) | 0.239 |
| ^1^The values represent n(%) for categorical variables | | | | |
| ^2^Wilcoxon rank sum test | | | | |
| *diff: difference between the corresponding left and right variables (e.g. SW_upper_diff = SW_RA - SW_LA) | | | | |

**Table S3.** Segmental body composition and bioimpedance variables

| **Variables** | **Total (n = 939)^1^** | **CN (n=673)^1^** | **MCI (n=266)^1^** | **p_value^2^** |
| --- | --- | --- | --- | --- |
| **SW_upper** |  |  |  | 0.568 |
| Mean (SD) | 1.687 (0.440) | 1.697 (0.442) | 1.684 (0.439) |  |
| **SW_lower** |  |  |  | 0.115 |
| Mean (SD) | 5.161 (1.179) | 5.262 (1.213) | 5.120 (1.164) |  |
| **SL_upper** |  |  |  | 0.580 |
| Mean (SD) | 2.167 (0.565) | 2.179 (0.568) | 2.163 (0.564) |  |
| **SL_lower** |  |  |  | 0.123 |
| Mean (SD) | 6.588 (1.509) | 6.714 (1.549) | 6.538 (1.491) |  |
| **Water_Lean_upper** |  |  |  | 0.254 |
| Mean (SD) | 0.779 (0.002) | 0.779 (0.002) | 0.779 (0.002) |  |
| **Water_Lean_lower** |  |  |  | **0.003** |
| Mean (SD) | 0.783 (0.002) | 0.784 (0.002) | 0.783 (0.002) |  |
| **ECW_ICW_upper** |  |  |  | **0.006** |
| Mean (SD) | 0.614 (0.010) | 0.615 (0.010) | 0.613 (0.010) |  |
| **ECW_ICW_lower** |  |  |  | **<0.001** |
| Mean (SD) | 0.662 (0.024) | 0.666 (0.024) | 0.660 (0.024) |  |
| **R_upper** |  |  |  | 0.197 |
| Mean (SD) | 206.7 (37.3) | 203.9 (35.8) | 207.8 (37.8) |  |
| **R_lower** |  |  |  | **0.017** |
| Mean (SD) | 145.8 (24.4) | 142.2 (23.2) | 147.2 (24.7) |  |
| **Xc_upper** |  |  |  | **0.003** |
| Mean (SD) | 29.4 (3.6) | 28.9 (3.6) | 29.6 (3.6) |  |
| **Xc_lower** |  |  |  | **<0.001** |
| Mean (SD) | 19.4 (3.9) | 18.6 (3.6) | 19.7 (3.9) |  |
| **PA_upper** |  |  |  | 0.282 |
| Mean (SD) | 5.2 (0.6) | 5.1 (0.6) | 5.2 (0.6) |  |
| **PA_lower** |  |  |  | **0.012** |
| Mean (SD) | 4.8 (0.8) | 4.7 (0.8) | 4.9 (0.8) |  |
| ^1^The values represent mean (SD) for continuous variables, and n (%) for categorical variables. The p-values for the continuous variables were obtained from an independent two sample t-test for normally distributed variables or Mann-Whitney-Wilcoxon rank sum test for non-normally distributed variables. For the categorical variables, the p-values were derived from the chi-squared test statistics or Fisher-exact test. | | | | |
| ^2^Wilcoxon rank sum test; Two Sample t-test | | | | |

**Table S4.** Estimated odds ratios and 95% confidence intervals derived from the logistic regression models for resistance, reactance, and phase angle variables at different frequencies.

| **Variables** | **Crude model** | | | **Adjusted model** | | |
| --- | --- | --- | --- | --- | --- | --- |
|  | **OR^1^** | **95% CI^1^** | **p-value^2^** | **OR^1^** | **95% CI^1^** | **p-value^2^** |
| **R_5kHz_upper** | 0.89 | 0.77, 1.02 | 0.100 | 0.81 | 0.63, 1.04 | 0.098 |
| **R_5kHz _lower** | 0.78 | 0.67, 0.90 | **<0.001** | 0.74 | 0.61, 0.90 | **0.002** |
| **R_50kHz _upper** | 0.90 | 0.78, 1.04 | 0.153 | 0.83 | 0.64, 1.07 | 0.144 |
| **R_50kHz _lower** | 0.81 | 0.70, 0.93 | **0.004** | 0.75 | 0.62, 0.92 | **0.004** |
| **R_250kHz _upper** | 0.91 | 0.79, 1.05 | 0.187 | 0.84 | 0.65, 1.08 | 0.174 |
| **R_250kHz _lower** | 0.82 | 0.71, 0.94 | **0.006** | 0.76 | 0.62, 0.92 | **0.005** |
| **Xc_5kHz _upper** | 0.81 | 0.70, 0.93 | **0.004** | 0.85 | 0.73, 1.00 | 0.053 |
| **Xc_5kHz _lower** | 0.72 | 0.62, 0.83 | **<0.001** | 0.79 | 0.66, 0.93 | **0.005** |
| **Xc_50kHz _upper** | 0.81 | 0.70, 0.94 | **0.004** | 0.86 | 0.73, 1.01 | 0.064 |
| **Xc_50kHz _lower** | 0.73 | 0.63, 0.84 | **<0.001** | 0.78 | 0.66, 0.92 | **0.004** |
| **Xc_250kHz _upper** | 0.85 | 0.74, 0.99 | **0.031** | 0.81 | 0.65, 1.01 | 0.065 |
| **Xc_250kHz _lower** | 0.76 | 0.66, 0.88 | **<0.001** | 0.80 | 0.68, 0.95 | **0.009** |
| **PA_5kHz _upper** | 0.90 | 0.78, 1.03 | 0.136 | 0.90 | 0.76, 1.07 | 0.248 |
| **PA_5kHz _lower** | 0.85 | 0.74, 0.99 | **0.031** | 0.91 | 0.76, 1.09 | 0.303 |
| **PA_50kHz _upper** | 0.89 | 0.77, 1.02 | 0.103 | 0.89 | 0.74, 1.06 | 0.195 |
| **PA_50kHz _lower** | 0.83 | 0.72, 0.96 | **0.013** | 0.89 | 0.74, 1.06 | 0.195 |
| **PA_250kHz _upper** | 0.86 | 0.75, 1.00 | **0.043** | 0.91 | 0.78, 1.07 | 0.244 |
| **PA_250kHz _lower** | 0.86 | 0.74, 0.99 | **0.035** | 0.92 | 0.78, 1.07 | 0.272 |
| ^1^OR = Odds Ratio, CI = Confidence Interval | | | | | | |
| ^2^P-value obtains from Wald test. Age, sex, GDS score, hyperlipidemia, diabetes and central nervous system disorders were controlled in the adjusted model. | | | | | | |
| ^*^Note: The results in this table were obtained from 938 participants, one observation fewer than the analysis in the main text due to data preprocessing. | | | | | | |
