# Supplementary figures and images for: Segmental Bioimpedance Variables in Association With Mild Cognitive Impairment
Source: Front Nutr. 2022 Jun 2;9:873623. doi: 10.3389/fnut.2022.873623 (PMC9201435; doi:10.3389/fnut.2022.873623)

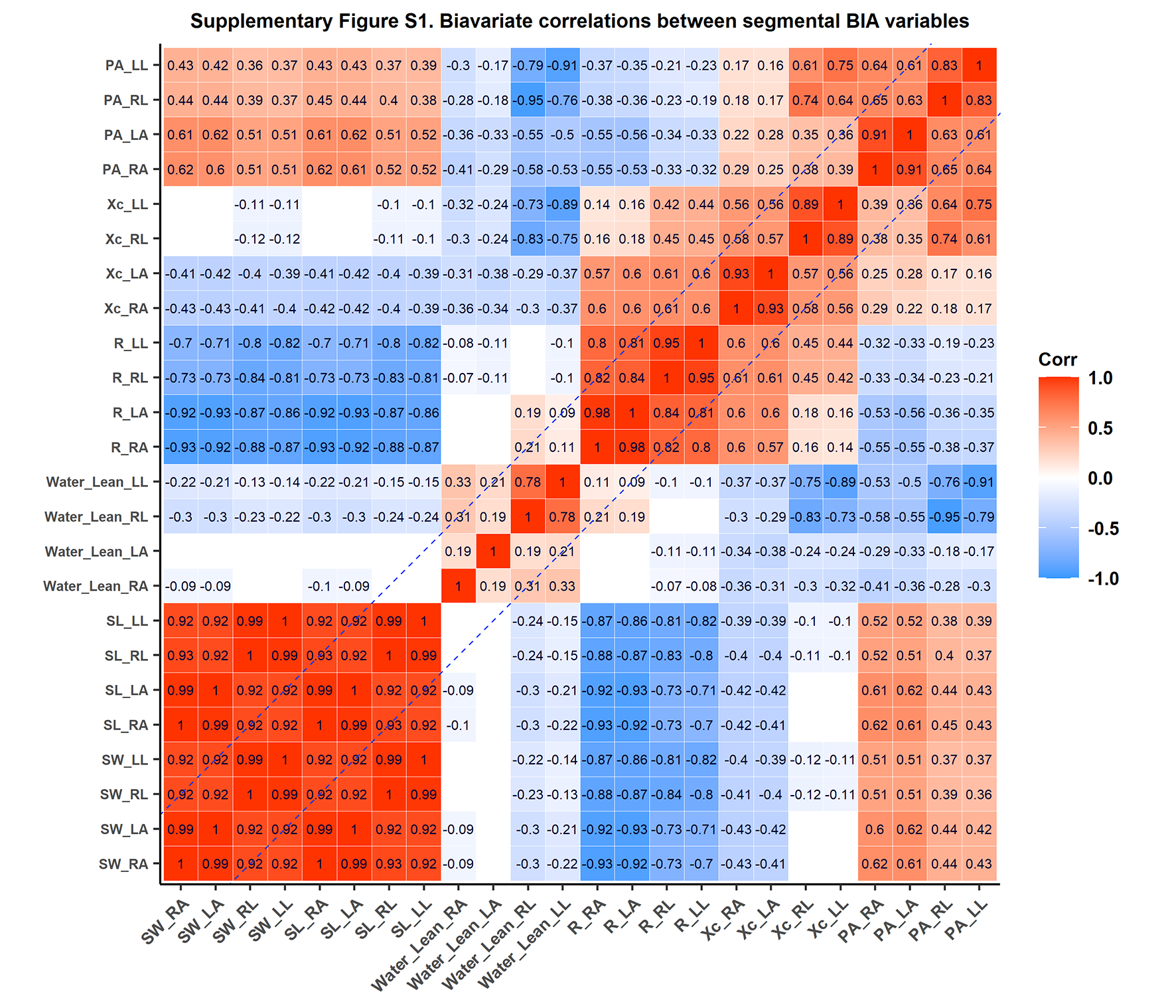

Supplement: Supplementary file 2 [file Image_1.TIFF]
